# Editorial: Bridging the gap between animal models and human addiction: exploring translational paradigms

**DOI:** 10.3389/fpsyg.2026.1866403

**Published:** 2026-06-02

**Authors:** Salvatore Campanella, Etienne Quertemont

**Affiliations:** 1 Laboratoire de Psychologie Médicale et d'Addictologie, ULB Neuroscience Institute (UNI), CHU Brugmann, Université Libre de Bruxelles (ULB), Brussels, Belgium; 2 Department of Psychology, University of Liège (ULiège), Liège, Belgium

**Keywords:** addiction, animal models, biomarkers, compulsive behaviors, cue reactivity, translational research

Decades of research have tried to identify the mechanisms underlying both the onset and the long-term maintenance of addictive behaviors. How and why can an initially arbitrary activity, such as watching TV series, drinking alcohol or gambling on sport events, become so central in an individual's life, and so compulsive that it will overtake all other activities? Classic substance-related addictions have primarily been conceptualized through their reinforcing properties on specific brain networks, that is, the mesolimbic circuit, and more precisely, the nucleus accumbens within the ventral striatum. Repeated exposure to an addictive substance promotes the encoding of its rewarding properties, acting as a reinforcement learning signal, increasing incentive salience and stimulating motor action. The transition from “simple impulses to consume” to “compulsive behaviors” has been associated with dysfunctions in (pre)frontal control systems, which are critical for future-oriented processes and for regulating current actions according to long-term goal-directed motivations. From this perspective, substance-related addictive behaviors have been described as a reinforced, learned automatic response (i.e., a habit) directed toward a highly salient activity, persisting despite outcome devaluation and goal-inappropriateness. Nevertheless, the mechanisms through which such habits are learned and operate still remain a topic of ongoing research and debate, with controversies regarding the exact contribution of (overestimated) habitual processes vs. (underestimated) contextual goal-directed processes.

Addiction research has traditionally involved animal research as well as experimental protocols performed on humans, with the goal of improving our understanding of the social, emotional, demographic, biological, and genetic mechanisms contributing to the onset and persistence of addictive behaviors. While such studies have clearly advanced the field, the dialogue between animal and human research remains challenging. This has led some authors to argue that “animal models of addiction have not served us well in understanding and treating addiction in humans” (Field and Kersbergen, 2020), while others still maintain that by allowing “a better characterization of the cerebral circuits involved, and the long-term modifications of the brain induced by addictive drugs administrations, … we might be able to find new treatments to normalize the altered brain homeostasis” (Cheron and Kerchove d'Exaerde, 2021). This ongoing debate underscores the need to develop and to implement new and original “translational” protocols, allowing researchers to improve the clinical relevance and accessibility of their experimental findings. This is essential for bridging the gap between bench and bedside and for fostering a more effective integration of preclinical and clinical research in the field of addiction.

Within this theoretical framework, the present Research Topic aims to highlight studies that combine well-established animal protocols with key concepts derived from human research. Rodriguez-Lausell et al. highlighted behavioral, circuit-level and molecular processes that contribute to renewal and reacquisition of drug-seeking behaviors across substances, processes which are key drivers of relapse in substance use disorders. They identify mechanistic gaps that currently limit intervention development. Bode et al. argued that paradigms assessing cue-control provide a valuable mechanistic bridge between basic learning theory and clinically relevant behavior. In the same translational perspective, Heck et al. emphasized the importance to bridge motivational and attentional perspectives across animal and human research, as motivational and attentional processes jointly determine how environmental cues influence behavior. Poorvi et al. provided rodent evidence indicating that methamphetamine and amphetamine induced enduring alterations in brain function, cognition and behavior, and stressing the importance of standardizing dosing regimens, withdrawal periods, and behavioral tasks to enhance translational relevance. Finally, Cofresi and Aponte Zabala reported findings from a pilot study in which *de novo* cue conditioning procedures from non-human animal models of alcohol cue reactivity were adapted for neuroimaging and psychophysiology experiments with human subjects, while Habelt proposed, based on a rat model of alcohol addiction, that event-related beta oscillations are an underexplored yet highly promising biomarker of cognitive function and recovery following therapeutic interventions.

Taken together, these contributions highlight the extreme complexity of the substance and non-substance addictive phenomenon. They emphasize the need for future studies to adopt a more integrative perspective, in order to better characterize the mechanisms through which addictive behaviors are acquired, expressed, and maintained. Ultimately, such translational approaches are expected to contribute to improved understanding, prevention, diagnosis, and treatment of addiction (Corley et al., 2024).
